# Effects of 12 weeks of upper‐body rowing exercise on autonomic cardiovascular control and vascular structure in spinal cord‐injured humans

**DOI:** 10.1113/EP092667

**Published:** 2025-08-12

**Authors:** Rasmus Kopp Hansen, Rasmus Bering, Claus Graff, Stefanos Volianitis, Uffe Laessoe, Afshin Samani, Ryan Godsk Larsen

**Affiliations:** ^1^ Respiratory and Critical Care Group, Department of Health Science and Technology, Faculty of Medicine Aalborg University Aalborg Denmark; ^2^ ExerciseTech, Department of Health Science and Technology, Faculty of Medicine Aalborg University Aalborg Denmark; ^3^ Department of Research and Development University College of Northern Jutland Aalborg Denmark; ^4^ Department of Health Science and Technology, Faculty of Medicine Aalborg University Aalborg Denmark; ^5^ Department of Sport Coaching, College of Sport Sciences Qatar University Doha Qatar

**Keywords:** autonomic cardiovascular function, exercise, heart rate variability, orthostatic intolerance, spinal cord injury, vascular adaptations

## Abstract

Spinal cord injury (SCI) is characterized by autonomic cardiovascular dysfunction that may contribute to the three‐ to fourfold greater risk of heart disease and stroke compared to non‐injured individuals. While exercise training elicits beneficial changes in autonomic function and vascular structure in healthy individuals, it is unclear if similar adaptations occur in individuals with SCI. Adults with chronic SCI (>1 year post injury) were randomized to 12 weeks of supervised upper‐body rowing exercise (UBROW; 3×/week; *n* = 8), adhering to current exercise guidelines, or control (CON; *n* = 9). Autonomic cardiovascular control was assessed by heart rate variability (HRV; electrocardiography) and blood pressure responses to a sit‐up test (finger plethysmography). Brachial (peripheral) and carotid (central) artery diameter and wall thickness (near‐ and far‐wall carotid intima–media‐thickness) were measured using high‐resolution ultrasound. All measurements were conducted at baseline, after 6 and 12 weeks. There was no effect of UBROW on time and frequency domain HRV or blood pressure responses to the sit‐up test (group‐by‐time interactions: *P* ≥ 0.28; effect sizes: η_p_^2^ ≤ 0.11). For UBROW, brachial artery diameter increased from 4.80 ± 0.72 mm at baseline to 5.08 ± 0.91 mm after 12 weeks (*P *< 0.05, η_p_^2^ = 0.27). Carotid artery dimensions did not change, and there were no correlations between changes (baseline–12 weeks) in brachial artery diameter and changes in HRV outcomes (*r* ≤ 0.40, *P* ≥ 0.14). While upper‐body rowing exercise enlarged brachial artery diameter, carotid artery dimensions and autonomic cardiovascular control did not change, suggesting local vascular remodelling, but no systemic vascular adaptations, in response to a supervised 12‐week exercise intervention in spinal cord‐injured humans.

## Abstract (highlights)

- **What is the central question of this study?**
  What are the effects of volitional upper‐body rowing exercise on autonomic cardiovascular control and vascular structure in individuals with chronic SCI?
- **What is the main findings and its importance?**
  This study demonstrates that 12 weeks of volitional upper‐body rowing exercise results in localized enlargement of the brachial artery diameter with no influence on carotid artery dimensions (diameter and wall thickness) or autonomic vascular control. These findings provide novel evidence of local (and not systemic) vascular adaptations in response to exercise training in individuals with SCI.

## 1 INTRODUCTION

Spinal cord injury (SCI) is an intricate neurological condition characterized not only by sensory and motor deficits below the neurological level of injury but also by autonomic cardiovascular dysfunction, which is considered to underpin the three‐ to fourfold greater risk of heart disease and stroke compared to non‐injured individuals (Cragg et al., 2013). Disruptions to the sympathetic arm of the autonomic nervous system (ANS) result from damage to the neural pathways between the medulla oblongata and the sympathetic pre‐ganglionic neurons (exiting the spinal cord between T1 and L2) at and below the level of injury (Krassioukov, 2009; Wulf & Tom, 2023) that is manifested as loss of descending supraspinal sympathetic outflow to the vasculature and heart. Depending on the injury level, SCI may lead to blood pressure instability, characterized by orthostatic hypotension (inability to tolerate postural stress due to insufficient sympathetic activation to maintain blood pressure), and/or autonomic dysreflexia (exaggerated elevation of blood pressure due to spinal‐mediated reflex activation of sympathetic neurons; Claydon & Krassioukov, 2006; Claydon et al., 2006). Long‐term blood pressure instability may lead to increased prevalence of cerebrovascular (Phillips et al., 2018) and cardiovascular diseases (Currie et al., 2019; Phillips et al., 2018). Moreover, SCI may also attenuate the cardiovascular response to acute exercise including reduced chronotropic response (Currie et al., 2015), and impaired redistribution of blood flow (Thijssen et al., 2009) that limits cardiac filling pressure and stroke volume (Jacobs et al., 2002), ultimately reducing exercise tolerance, with adverse consequences for exercise training adaptations (Myers et al., 2007). It has also been suggested that heart rate variability (HRV), a measure of autonomic modulation of heart rate (Shaffer & Ginsberg, 2017), may be altered by SCI (Hodgkiss et al., 2025). Specifically, studies have reported reduced low‐frequency (LF) power HRV in cervical SCI (Buker et al., 2018; Bunten et al., 1998; Claydon & Krassioukov, 2008; Rodrigues et al., 2016) and reduced high‐frequency (HF) power HRV in thoracic SCI (Claydon & Krassioukov, 2008), which may contribute to elevated mortality and greater risk of cardiovascular disease (Thayer et al., 2010).

In healthy individuals, whole‐body exercise training improves autonomic cardiovascular control, including enhanced parasympathetic control of heart rate (Carter et al., 2003), and orthostatic tolerance (Wieling et al., 2002) although the latter is not a universal finding (Levine, 1993). Whilst research into autonomic disorders after SCI has received increasing clinical priority during the last years (Hou & Rabchevsky, 2014), few studies have explored the potential modulating effects of exercise training on autonomic cardiovascular control, with even fewer investigating the effects induced by volitional upper‐body exercise interventions (i.e. in the absence of electrical stimulation or assisted treadmill walking) (Alajam et al., 2019; Vivodtzev & Taylor, 2021). Recently, Dorey et al. (2024) indicated improved parasympathetic regulation following an arm‐cranking exercise intervention in individuals with high‐level SCI, by showing enhanced cardiovagal baroreflex sensitivity alongside reductions in HF power HRV responses to a sit‐up test. In contrast, no effects were reported on blood pressure responses to the sit‐up test or incidence of orthostatic hypotension, consistent with an absence of exercise‐induced changes in sympathetic‐mediated peripheral vascular resistance (Dorey et al., 2024). There is some evidence that 6 months of functional electrical stimulation (FES) cycling enhances catecholamine response to maximal arm‐cranking exercise in individuals with SCI (Bloomfield et al., 1994). However, the value of plasma noradrenaline as an indicator of autonomic sympathetic activity in this population is questioned (Bloomfield et al., 1994). Taken together, it is therefore currently unclear if individuals with SCI experience exercise‐induced improvements in HRV and orthostatic tolerance, or whether such adaptations are prevented by the unique autonomic disruption commonly observed in this population.

Impaired vascular function and structure are common characteristics in people with SCI, which also may contribute to the elevated cardiovascular disease risk in this population (West, Alyahya et al., 2013). For instance, thickening of the carotid artery wall, a validated surrogate marker of atherosclerosis (De Groot et al., 2004), has been reported in individuals with SCI compared to healthy controls (Matos‐Souza et al., 2009). While cross‐sectional studies suggest lower wall thickness and wall‐to‐lumen ratio of both central (carotid) and peripheral (brachial) arteries in exercising athletes with SCI compared with SCI controls (Rowley et al., 2012), longitudinal studies using volitional upper‐body exercise training to study adaptations in arterial function and structure are inconclusive (Alrashidi et al., 2021; Hansen et al., 2023; Totosy de Zepetnek et al., 2015; Williams et al., 2021). Studies using exercise interventions adhering to the previous (i.e. 2 × 40 min moderate to vigorous intensity per week; Ginis et al., 2011) or the current guidelines (i.e. 3 × 30 min moderate to vigorous intensity per week; Martin Ginis et al., 2018) showed no effect on carotid pulse wave velocity (Alrashidi et al., 2021), or brachial artery flow‐mediated dilatation (Hansen, Samani, Laessoe et al., 2023; Totosy de Zepetnek et al., 2015). In contrast, previous work from our laboratory demonstrated that 12 weeks of upper‐body rowing exercise (3 × 30 min/week) increased brachial artery resting diameter in people with SCI (Hansen, Samani, Laessoe et al., 2023). However, it is unclear if the enlargement of brachial artery dimension could be attributed to concurrent changes in sympathetic nervous activity (i.e. reduced sympathetic tone) rather than structural remodelling (Chacon & Chopek, 2023; Hansen, Samani et al., 2023). Also, it is unclear whether adaptations are confined to the arteries supplying the exercising upper limbs (e.g. brachial artery), or if systemic (i.e. central) adaptations occur in response to upper‐body exercise training.

This study evaluated the effects of 12 weeks of upper‐body rowing exercise on measures of autonomic cardiovascular control and vascular structure in individuals with SCI. Specifically, we assessed HRV and orthostatic tolerance (measured as the blood pressure responses to a sit‐up test), and lumen diameter and wall thickness of peripheral (brachial) and central (carotid) arteries. Furthermore, we explored potential associations between changes (baseline–12 weeks) in lumen diameter and changes in HRV parameters.

## 2 METHODS

### 2.1 Study design and participants

The data presented in this study were collected as part of a randomized controlled trial (clinical trial registration: NCT04390087) approved by the North Denmark Region Committee on Health Research Ethics (N‐20190053). A detailed study protocol has previously been published (Hansen et al., 2020). Briefly, participants were randomized to either a 12‐week upper‐body rowing exercise intervention (UBROW, *n* = 8) or a control group (CON, *n* = 9) (Table 1). The study was conducted in accordance with ethical principles for studies involving human participants set out in the *Declaration of Helsinki*, and all participants received verbal and written information about the study before they signed consent to participate in the study.

**TABLE 1:** Baseline participant characteristics.

	CON (*n* = 9)	UBROW (*n* = 8)
Sex (M/F)	5/4	5/3
Age (years)	50 ± 12	50 ± 4
Height (m)	1.76 ± 0.11	1.75 ± 0.15
Body mass (kg)	81.0 ± 18.1	86.7 ± 12.5
BMI (kg/m^2^)	26.3 ± 6.1	28.6 ± 5.7
Injury*		
AIS A–B	5	4
AIS C–D	3	4
NLI	C4–L3	C6–L2
TSI (years)	24 ± 10	36 ± 11

Values are numbers or means ± SD. *One participant in CON with unknown AIS, NLI and TSI. Abbreviations: AIS, American Spinal Injury Association Impairment Scale; BMI, body mass index; CON, control group; F, female; M, male; NLI, neurological level of injury; TSI, time since injury; UBROW, upper‐body rowing exercise group.

Participants were recruited based on the following inclusion criteria: aged between 18 and 70 years; being wheelchair dependent; with a traumatic or non‐traumatic chronic SCI (≥1 year post‐injury); motor‐complete or incomplete SCI (American Spinal Injury Association Impairment Scale (AIS) A–D, determined via the International Standards for Neurological Classification of Spinal Cord Injury; Kirshblum et al., 2011). There were no specific requirements for the participants’ neurological level of injury (NLI), other than sufficient arm flexor function to allow upper‐body rowing. Individuals who self‐reported active current medical issues, including urinary tract infections, pressure sores, cardiovascular contraindications for exercise testing, diagnosed diabetes, or any other disease that restricted exercise, were excluded.

### 2.2 Exercise training protocol

Participants in UBROW completed 12 weeks of three weekly supervised sessions of 30 min moderate‐to‐vigorous intensity indoor rowing exercise, complying with current exercise guidelines for adults with SCI (Martin Ginis et al., 2018). Details about the rowing intervention have been published elsewhere (Hansen et al., 2022, 2020). Briefly, the rowing exercise was performed on a commercially available rowing ergometer (Concept2 Indoor Rower D PM5, Morrisville, VT, USA) adapted for wheelchair users by an Adapt2Row unit. Exercise intensity of each training session was prescribed based on a rating of perceived exertion (RPE) using the Borg 6–20 RPE scale (Borg, 1970), aiming at intensities from moderate (RPE: 12–13) to vigorous (RPE: 14–17) (Hutchinson & Goosey‐Tolfrey, 2022). The use of RPE to control moderate and vigorous intensity has been validated in individuals with SCI (Goosey‐Tolfrey et al., 2010). Heart rate was also recorded continuously using a chest belt (Movense Smart Sensor, Suunto, Vantaa, Finland). Participants randomized to CON were requested to maintain their normal lifestyle throughout the 12 weeks. All exercise sessions were supervised by health professionals.

### 2.3 Procedures/experimental protocol

Assessments of HRV, orthostatic tolerance, and peripheral and central artery adaptations (as outlined below) were performed before (baseline), midway (6 wk), and immediately after the end of the 12‐week intervention (12 wk) to track the nature and time course of adaptations over the training period. All experimental procedures were performed in the Exercise Laboratory at Aalborg University. Participants were asked not to change their dietary habits during the 12‐week intervention period. Prior to testing, participants were instructed to abstain from any vigorous‐intensity exercise for at least 24 h; caffeine, alcohol, polyphenols and vitamin C for 12 h; and food for at least 3 h. Prior to both testing and exercise sessions, participants were asked to empty their bladder to minimize the risk of reflex sympathetic activation. All tests were conducted at least 24 h after the last exercise bout to avoid interference from any resilient fatigue and ensure consistency in our assessments. None of the participants were smokers and none of the female participants were eumenorrheic.

### 2.4 Heart rate variability

After arrival at the laboratory, body characteristics were measured. Subsequently, participants were fitted with a single‐lead ECG and rested for 10–15 min in the supine position in a quiet, temperature‐controlled room (range: 22.1–24.2°C). Hereafter, the room was darkened, and a 5‐min ECG was acquired with continuous measurement of heart rate while participants remained relaxed and breathed spontaneously. Cardiac ECG was sampled at 5000 Hz (LabScribe V.4, iWorx, Dover, NH, USA) and exported to dedicated software (Kubios HRV Standard V.3.2.0; Kuopio, Finland) for ECG filtering and R‐wave detection and analyses of resting HRV. Time and frequency domain analysis of the R–R interval was performed and reported in accordance with guidelines from the European Society of Cardiology and Heart Rhythm Society (Shaffer & Ginsberg, 2017; Task Force of The European Society of Cardiology & The North American Society of Pacing & Electrophysiology, 1996). In the frequency domain, analyses of low‐frequency (LF) power and high‐frequency (HF) power were performed, with time‐domain parameters including mean heart rate and the root mean square of successive RR interval differences (RRMSD).

### 2.5 Orthostatic tolerance (sit‐up test)

The blood pressure response to an orthostatic challenge was evaluated using a standardized sit‐up test (Claydon & Krassioukov, 2006). Beat‐to‐beat arterial blood pressure and heart rate were recorded using finger photoplethysmography (Finometer, Finapres Medical Systems BV, Enschede, The Netherlands). A height sensor was fixed on one arm at the level of the heart to automatically correct for hydrostatic pressure influences. Continuous non‐invasive blood pressure monitoring was performed for 20 min. After 10 min of recordings at supine rest, participants were passively moved into an upright seated position with their legs hanging free from the bed at an angle of 90°. Participants were instructed not to assist in this ‘sit‐up’ procedure. The seated position was maintained for 10 min while participants were closely monitored for any severe symptoms of autonomic dysreflexia or presyncope, that would terminate the test (*n* = 0). The mean change (Δ) in systolic blood pressure (SBP) and diastolic blood pressure (DBP) was defined as the difference between mean seated and supine values (Mean ΔBP = BP_seated_ − BP_supine_). We also averaged seated blood pressures into 30‐s intervals for calculation of the maximum change in blood pressure defined as the difference between the lowest seated blood pressures (minimum 30‐s average) and the mean supine blood pressures (Max ΔBP = BP_seated_ − BP_supine,min_). The presence of orthostatic hypotension (OH) was defined as a decrease in SBP of ≥20 mmHg or ≥10 mmHg in DBP upon moving from the supine to seated position (Freeman et al., 2011).

### 2.6 Vascular ultrasound imaging

After at least 10 min of supine rest, artery diameter (brachial and common carotid) and wall thickness (common carotid) were measured by the same trained sonographer. A 10 MHz multifrequency linear‐array ultrasound probe (9L‐D) coupled to a high‐resolution ultrasound machine (LOGIQ S8 XDclear, GE Healthcare, Chicago, IL, USA) was used to image both arteries, with the ultrasound parameters set to optimize the longitudinal brightness‐mode images of the lumen/arterial wall interface. Assessment of central artery diameter and wall thickness (common carotid artery) was performed with the participant supine and the neck slightly extended to allow scanning of the artery. Recordings were performed unilaterally on the right carotid artery (*n* = 14), except for one participant who was scanned on the left artery due to better image quality. Importantly, all participants were consistently scanned on the same side across study visits. Images of the common carotid artery were made approximately 1–2 cm proximal to the bifurcation. Assessment of peripheral artery diameter was performed on the right brachial artery with the participants’ arm extended and positioned at an angle of ∼80° from the torso on a height‐adjustable table. The artery was scanned in the distal one‐third of the upper arm. Ultrasound imaging settings for both arteries were kept constant within participants across the three study visits.

### 2.7 Analysis of conduit artery diameter and wall thickness

Resting brachial artery diameter (M‐line to M‐line) was analysed off‐line frame‐by‐frame using semi‐automated edge‐detecting and wall‐tracking software (Version 6.0, Medical Imaging Applications LLC, Coralville, IA, USA), which is largely independent of investigator bias. A 30‐s image of the artery was acquired to measure resting artery diameter. Wall thickness, measured by near and far‐wall carotid intima–media‐thickness (CIMT), was defined as the distance from the media–adventitia border (M‐line) to the lumen–intima border (I‐line). The region of interest (ROI) was defined with a minimum length of 1 cm and placed where clear borders of adventitia, media and intima were evident at both vessel walls. For the repeated analyses (6 wk, 12 wk) the ROI was placed as close to the baseline sequence as the image quality allowed. To account for differences in artery diameter, we also calculated the wall‐to‐lumen ratio (Thijssen et al., 2013).

### 2.8 Cardiopulmonary exercise test

As detailed in the published protocol paper (Hansen et al., 2020) and studies reporting pre–post changes in cardiorespiratory fitness (Hansen, Laessoe et al., 2023; Hansen, Samani, Laessoe et al., 2023), a cardiopulmonary exercise test was performed on an arm‐crank ergometer (Monark 881E, Vansbro, Sweden) to determine peak exercise capacity, including peak oxygen uptake (${\dot{V}}_{O_{2}\mathrm{peak}}$), and peak power output (PPO). Considering that attenuated peak heart rate may be secondary to impaired sympathetic function, we interpreted heart rate (HR_peak_) as a gross ‘functional surrogate measure’ of sympathetic function (Currie et al., 2015). Briefly, after a warm‐up with zero resistance (Eerden et al., 2018; Hansen et al., 2020), the exercise test began at an individualized starting workload (0–50 W) followed by increments of 3.5 W (tetraplegics) or 7 W (paraplegics) each minute until volitional fatigue, defined as the inability to maintain cadence >60 rpm despite verbal encouragement. The individual starting load was determined based on training history and anticipated physical capacity, with the aim of reaching exhaustion within 8–12 min (Hansen et al., 2020). The starting workload and increments were kept constant within participants across laboratory visits. Heart rate was recorded continuously during the test using a chest belt (Movense Smart Sensor, Suunto, Vantaa, Finland), with HR_peak_ defined as the highest heart rate obtained during the test. Verbal encouragement was provided during the test.

### 2.9 Prior publications from the UBROW trial

Prior manuscripts from the UBROW intervention reported the feasibility and acceptability of adapted rowing exercise (i.e. upper‐body rowing) (Hansen et al., 2022), and demonstrated that 12 weeks of upper‐body rowing evokes improvements in cardiorespiratory fitness without impacting traditional cardiometabolic risk factors (including the primary outcome, fasting insulin), or flow‐mediated dilatation (Hansen, Samani, Laessoe et al., 2023). A follow‐up study has also been published addressing the longer‐term effects of the UBROW intervention on cardiorespiratory fitness, leisure‐time physical activity and cardiometabolic health (Hansen, Laessoe et al., 2023). Here, we uniquely report measures of autonomic cardiovascular control (HRV, orthostatic tolerance) and carotid artery diameter and wall thickness, which were secondary outcomes from the UBROW trial (Hansen et al., 2020). Pre–post intervention changes in brachial artery diameter have previously been reported (Hansen, Samani, Laessoe et al., 2023), but these data will also be presented here to support the second aim of the present report.

### 2.10 Statistical analysis

Normal distribution of data was assessed by visual inspection of the data and by the Shapiro–Wilk test. Non‐normally distributed HRV data were log‐transformed, checked again for normality, and then used as continuous log‐transformed variables in the statistical analyses. Responses to the intervention were analysed by a two‐way mixed model ANOVA with repeated measures, with time (baseline, 6 wk, 12 wk) and group (UBROW, CON) as within and between‐group factors, respectively. In the case of missing data (one time point for HRV outcomes (*n* = 1) and the sit‐up test (*n* = 1)), analyses were made using mixed‐effects models, with group and time as fixed factors, and subject as random factor. Where significant interaction effects were observed, pairwise comparisons were performed using Holm–Šidák *post hoc* test. Pearson's correlations were used to explore potential associations between changes (baseline to 12 wk) in lumen diameter and changes in HRV parameters. Categorical data (incidence of OH) were analysed by Fisher's exact test due to sample size. Statistical analyses were conducted in SPSS version 29 (IBM Corp., Armonk, NY, USA) and GraphPad Prism version 10 (GraphPad Software, Boston, MA, USA). Partial eta squared (η_p_^2^) was computed to determine standardized effect size and defined as small (η_p_^2^ = 0.01), medium (η_p_^2^ = 0.06) and large (η_p_^2^ = 0.14) (Lakens, 2013). Statistical significance was accepted at α* <* 0.05. Data are expressed as (non‐transformed) means ± SD, unless otherwise stated.

## 3 RESULTS

The number of recruited participants for this study was reduced due to the COVID‐19 pandemic. Consequently, 18 participants (with *n* = 1 excluded prior to randomization) were recruited for this study compared to the 30 participants stated in the protocol. Out of the 17 participants randomized, *n* = 15 completed the study. All subjects in UBROW (*n* = 8) completed the 12‐week intervention. Over the 12 weeks, the eight participants rowed an average distance of 4.1 ± 1.2 km per session, with power output, RPE and %HR_peak_ averaging 42 ± 21 W, 15.8 ± 0.7 and 83 ± 3%, respectively, across all sessions.

### 3.1 Effects of exercise training on the vasculature

**FIGURE 1:**
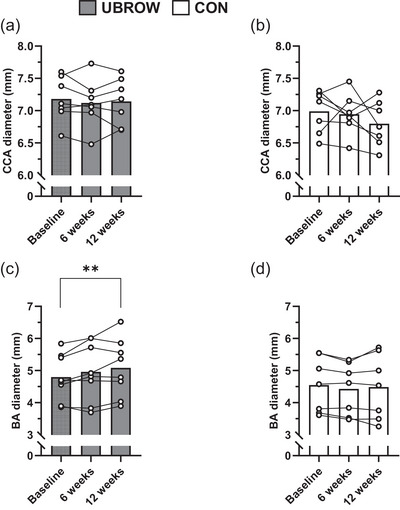
Individual and mean data for common carotid artery (CCA; a, b) and brachial artery (BA; c, d) lumen diameter in CON and UBROW, at baseline, after 6 weeks (midway) and 12 weeks (post‐intervention). **Significantly greater than baseline (*P *< 0.01).

For one participant in UBROW, clear vascular borders of the carotid artery could only be imaged at 6 wk. Therefore, this participant was excluded from analyses, so that comparisons for carotid artery diameter and wall thickness are based on *n* = 14. Training had no effect on carotid artery diameter (Figure 1a, b), CIMT near‐wall, CIMT far‐wall or wall‐to‐lumen ratio (all *P* > 0.05, Table 2), although an effect of group was found for CIMT far‐wall and wall‐to‐lumen ratio (Table 2). In contrast, there was a significant time‐by‐group interaction for brachial artery diameter with a large effect size (*P* = 0.016, η_p_^2^ = 0.272, Table 2). Specifically, exercise resulted in an increase in brachial artery diameter from baseline (4.80 ± 0.72 mm) to 12 wk (5.08 ± 0.91 mm; *P *< 0.01), with no changes at 6 wk (4.96 ± 0.91 mm, *P* = 0.166), and no changes in CON (*P* ≥ 0.536) (Figure 1c, d). There were no significant correlations between changes (baseline–12 wk) in carotid and brachial artery diameter and changes (baseline–12 wk) in HRV outcomes (Table 3).

**TABLE 2:** HRV and vascular outcomes at baseline, midway (6 wk) and post‐intervention (12 wk) in CON and UBROW.

		Baseline	Midway (6 wk)	Post‐intervention (12 wk)	Effect size η_p_^2^	*P*
Frequency domain HRV						
LF power (ms^2^)	CON	642 (−81 to 1365)	509 (−214 to 1231)	1070 (348–1793)	0.040	Time × group: *P* = 0.711 Time: *P* = 0.474 Group: *P* = 0.775
	UBROW	697 (21–1373)	395 (216–574)^a^	453 (−223 to 1129)		
HF power (ms^2^)	CON	2056 (−330 to 4441)	1812 (−573 (4197)	1822 (−564 to 4207)	0.040	Time × group: *P* = 0.682 Time: *P* = 0.734 Group: *P* = 0.527
	UBROW	641 (−1590 to 2872)	185 (−2230 to 2601)^a^	308 (−1923 to 2539)		
LF/HF ratio	CON	1.91 (0.59–3.23)	1.68 (0.36–2.99)	2.17 (0.85–3.49)	0.000	Time × group: *P* = 0.998 Time: *P* = 0.637 Group: *P* = 0.425
	UBROW	2.41 (1.17–3.64)	2.47 (1.14–3.79)^a^	2.27 (1.03–3.50)		
Time domain HRV						
Mean HR (bpm)	CON	63 (55–70)	63 (55–71)	62 (55–70)	0.003	Time × group: *P* = 0.833 Time: *P* = 0.899 Group: *P* = 0.687
	UBROW	61 (54–68)	64 (58–71)^a^	62 (55–69)		
RMSSD (ms)	CON	52.3 (8.4–96.1)	51.7 (7.8–95.5)	54.5 (10.6–98.3)	0.036	Time × group: *P* = 0.578 Time: *P* = 0.918 Group: *P* = 0.551
	UBROW	34.9 (−6.2 to 75.9)	23.3 (−22.7 to 69.3) ^a^	25.2 (−15.8 to 66.2)
Vascular imaging						
BA diameter (mm)	CON	4.55 (3.84–5.26)	4.43 (3.72–5.14)	4.49 (3.78–5.19)	0.272	Time × group: *P* = 0.016 Time: *P* = 0.158 Group: *P* = 0.321
	UBROW	4.80 (4.13–5.46)	4.96 (4.30–5.62)	5.08 (4.42–5.75)		
CCA diameter (mm)	CON	6.99 (6.71–7.27)	6.94 (6.66–7.23)	6.80 (6.50–7.09)	0.070	Time × group: *P* = 0.418 Time: *P* = 0.273 Group: *P* = 0.180
	UBROW *n* = 7	7.18 (6.90–7.46)	7.12 (6.83–7.41)	7.14 (6.85–7.44)		
CIMT near‐wall (mm)	CON	0.73 (0.66–0.79)	0.73 (0.68–0.78)	0.71 (0.65–0.78)	0.024	Time × group: *P* = 0.749 Time: *P* = 0.952 Group: *P* = 0.527
	UBROW	0.69 (0.63–0.76)	0.70 (0.65–0.76)	0.71 (0.65–0.78)		
CIMT far‐wall (mm)	CON	0.65 (0.60–0.70)	0.61 (0.58–0.65)	0.60 (0.56–0.65)	0.199	Time × group: *P* = 0.069 Time: *P* = 0.174 Group: ***P* = 0.025**
	UBROW *n* = 7	0.56 (0.51–0.61)	0.59 (0.55–0.62)	0.55 (0.50–0.60)		
CCA wall‐to‐lumen ratio^b^	CON	0.094 (0.086–0.102)	0.088 (0.083–0.094)	0.089 (0.083–0.095)	0.233	Time × group: *P* = 0.134 Time: *P* = 0.531 Group: ***P* = 0.009**
	UBROW *n* = 7	0.078 (0.070–0.086)	0.083 (0.077–0.088)	0.077 (0.071–0.083)		
CPET						
HR_peak_ (bpm)	CON	143 (116–170)	141 (116–167)	138 (110–165)	0.003	Time × group: *P* = 0.958 Time: *P* = 0.246 Group: *P* = 0.819
	UBROW	146 (121–171)	146 (123–169)	142 (116–168)		

Data are presented as means (95% Confidence Intervals). CON n = 7; UBROW n = 8, unless otherwise stated. ^a^ *n* = 7 in UBROW due to missing value for one subject. ^b^Wall‐to‐lumen ratio was calculated based on far‐wall CIMT. BA, brachial artery; CCA, common carotid artery; CIMT, carotid intima‐media‐thickness; CPET, cardiopulmonary exercise test; HF, high frequency; HR_peak_, heart rate peak; HRV, heart rate variability; LF, low frequency; Mean HR, mean heart rate; RMSSD, root mean square of successive RR interval differences.

**TABLE 3:** Correlation coefficients between changes (Δ) from baseline to 12 weeks in carotid and brachial artery diameter and Δ in HRV outcomes.

	vs ΔBA diameter (*n* = 15)		vs ΔCCA diameter (*n* = 14)	
	Pearson *R* (95% CI)	*P*	Pearson *R* (95% CI)	*P*
ΔRMSSD log10	0.35 (−0.20 to 0.73)	0.199	0.17 (−0.40 to 0.64)	0.567
ΔMean HR log10	−0.11 (−0.59 to 0.42)	0.688	0.16 (−0.40 to 0.64)	0.577
ΔLF power log10	0.27 (−0.29 to 0.69)	0.338	0.30 (−0.28 to 0.72)	0.300
ΔHF power log10	0.40 (−0.14 to 0.76)	0.136	0.36 (−0.22 to 0.75)	0.213
ΔLF/HF ratio log10	−0.15 (−0.62 to 0.39)	0.585	−0.02 (−0.55 to 0.51)	0.935

Abbreviations: BA, brachial artery; CCA, common carotid artery; HF, high frequency; mean HR, mean heart rate; LF, low frequency; RMSSD, root mean square of successive RR interval differences.

### 3.2 Effects of exercise training on exercise peak heart rate

There were no interaction or main effects observed for HR_peak_, and the effect size was small (η_p_^2^ = 0.003) (Table 2).

### 3.3 Effects of exercise training on heart rate variability

**FIGURE 2:**
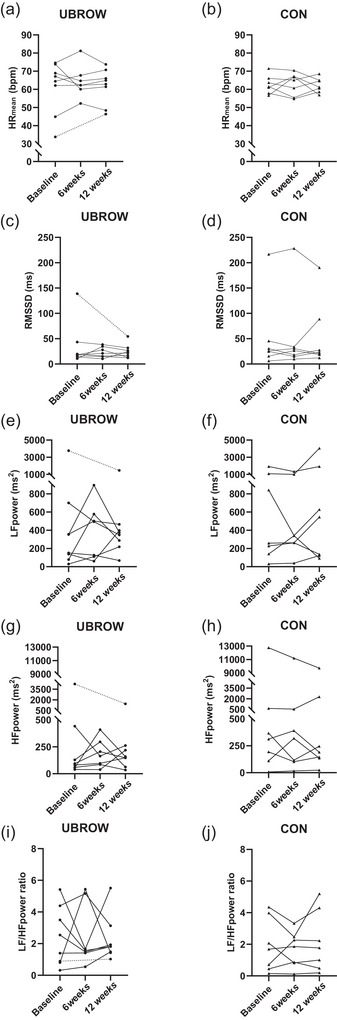
Individual data for time and frequency domain measures of heart rate variability (HRV) in CON and UBROW, at baseline, after 6 weeks (midway) and 12 weeks (post‐intervention). (a, b) Mean heart rate (HR_mean_); (c, d) root mean squared of successive differences in RR‐interval (RMSSD); (e, f) low‐frequency power of the HRV spectrum (LF power); (g, h) high‐frequency power of the HRV spectrum (HF Power); (i, j) low‐frequency to high‐frequency power ratio (LF/HF ratio). The broken line in UBROW represents data for the participant (NLI: C6, AIS: B) with missing ECG data at 6 weeks.

Due to insufficient quality of the ECG, HRV data are missing from one subject in UBROW at 6 wk and the HRV analysis is therefore based on mixed‐effect models rather than two‐way ANOVA. For the time domain analysis, we found no influence of exercise training on mean heart rate (η_p_^2^ = 0.003) or RMSSD (η_p_^2^ = 0.036) (interaction effects *P* ≥ 0.578; Table 2, Figure 2a–d). In the frequency domain, neither LF power, nor HF power nor LF/HF ratio was affected by exercise training (interaction effects *P* ≥ 0.682; η_p_^2^: 0.00–004; Table 2, Figure 2e–j).

### 3.4 Effects of exercise training on blood pressure and orthostatic tolerance

One participant in CON exhibited a disturbed finger photoplethysmography signal due to peripheral vasoconstriction (cold fingers) at baseline and 6 wk, and we therefore excluded this participant from the analysis (i.e. *n* = 6 in CON). There were also missing data from one participant in UBROW at baseline (due to cold fingers); however, as the data from the remaining assessments were of good quality, we included the available data from this participant in mixed‐effect model analyses. Arterial blood pressure and heart rate from the orthostatic challenge are reported in Table 4. Training did not affect supine or seated blood pressures (all time × group interactions, *P* > 0.05). However, a main effect of time was found for supine DBP, mean seated SBP and DBP, and the lowest seated SBP and DBP, reflecting an overall decline in these blood pressure variables over time (Table 4). There were no significant interaction effects for changes in blood pressure, with small (η_p_^2^: 0.023–0.025) and medium (η_p_^2^: 0.100–0.106) effect sizes (Table 5). Similarly, there were no significant differences between the groups in the incidence of OH at any time point, when calculated based on either mean (*P* ≥ 0.429) or max (all *P* = 1.000) changes in blood pressure (Table 5).

**TABLE 4:** Blood pressure values before and after an orthostatic challenge at baseline, midway (6 wk) and post‐intervention (12 wk) in CON and UBROW.

		Baseline	Midway (6 wk)	Post‐intervention (12 wk)	Effect size η_p_^2^	*P*
SBP supine (mmHg)	CON	130 (107–153)	136 (109–163)	124 (104–143)	0.097	Time × group: *P* = 0.277 Time: *P* = 0.098 Group: *P* = 0.690
	UBROW	137 (116–158)^a^	134 (111–158)	131 (114–149)		
Mean SBP seated (mmHg)	CON	138 (111–165)	138 (109–168)	124 (95–153)	0.012	Time × group: *P* = 0.772 Time: *P* = 0.016 Group: *P* = 0.511
	UBROW	144 (119–168)^a^	148 (123–174)	139 (114–164)		
Lowest SBP seated (mmHg)	CON	124 (94–153)	127 (97–156)	107 (78–137)	0.052	Time × group: *P* = 0.512 Time: *P* = 0.011 Group: *P* = 0.817
	UBROW	118 (91–145) ^a^	130 (105–156)	117 (92–143)		
DBP supine (mmHg)	CON	79 (68–90)	76 (64–89)	69 (60–79)	0.074	Time × group: *P* = 0.470 Time: *P* = 0.046 Group: *P* = 0.541
	UBROW	81 (71–91)^a^	77 (67–88)	77 (68–85)		
Mean DBP seated (mmHg))	CON	83 (68–98)	80 (66–94)	68 (53–84)	0.062	Time × group: *P* = 0.444 Time: *P* = 0.002 Group: *P* = 0.392
	UBROW	87 (73–101)^a^	87 (74–99)	80 (66–93)		
Lowest DBP seated (mmHg)	CON	72 (56–88)	73 (59–87)	57 (40–74)	0.099	Time × group: *P* = 0.550 Time: *P* = 0.010 Group: *P* = 0.500
	UBROW	73 (58–88)^a^	78 (66–91)	68 (53–83)		
HR supine (bpm)	CON	65 (55–75)	64 (56–73)	63 (58–68)	0.106	Time × group: *P* = 0.280 Time: *P* = 0.820 Group: *P* = 0.600
	UBROW	59 (50–68)^a^	62 (55–70)	64 (59–68)		
Mean HR seated (bpm)	CON	74 (65–83)	71 (64–78)	70 (62–78)	0.080	Time × group: *P* = 0.399 Time: *P* = 0.374 Group: *P* = 0.202
	UBROW	65 (57–74)^a^	68 (62–78)	65 (58–71)		
Lowest HR seated (bpm)	CON	67 (57–76)	65 (57–72)	64 (55–72)	0.043	Time × group: *P* = 0.604 Time: *P* = 0.438 Group: *P* = 0.458
	UBROW	61 (52–70)^a^	62 (55–69)	61 (54–68)		

Data are presented as means (95% confidence Intervals). ^a^ *n* = 7 in UBROW due to missing value for one subject. Note that for all blood pressure values, *n* = 6 in CON. CON, control group; DBP, diastolic blood pressure; HR, heart rate; SBP, systolic blood pressure; UBROW, upper‐body rowing exercise group.

**TABLE 5:** Mean and maximum changes in arterial blood pressure and incidence of OH at baseline, midway (6 wk) and post‐intervention (12 wk) in CON and UBROW.

		Baseline	Midway (6 wk)	Post‐intervention (12 wk)	Effect size η_p_^2^	*P*
Mean changes						
Mean ΔSBP (mmHg)	CON	8.2 (−7.7 to 24.0)	2.2 (−14.6 to 18.9)	0.8 (−14.7 to 16.3)	0.106	Time × group: *P* = 0.298 Time: *P* = 0.610 Group: *P* = 0.512
	UBROW	6.6 (−8.1 to 21.3)^a^	13.9 (−0.6 to 28.4)	7.8 (−5.6 to 21.1)		
Mean ΔDBP (mmHg)	CON	4.2 (−6.1 to 14.4)	4.0 (−6.8 to 14.8)	−1.2 (−10.6 to 8.2)	0.025	Time × group: *P* = 0.789 Time: *P* = 0.110 Group: *P* = 0.511
	UBROW	5.7 (−3.8 to 15.2)^a^	9.1 (−0.2 to 18.5)	3.0 (−5.2 to 11.2)		
Incidence of OH (*n*/total sample (%))	CON	1/6 (16.7%) *P* = 1.00	2/6 (33.3%) *P* = 0.165	1/6 (16.7%) *P* = 0.429		
	UBROW	1/7 (14.3%)	0/8 (0%)	0/8 (0%)		
Max changes						
Max ΔSBP (mmHg)	CON	−6.2 (−25.5 to 13.2)	−9.3 (−28.4 to 9.7)	−16.3 (−36.4 to 3.8)	0.100	Time × group: *P* = 0.283 Time: *P* = 0.321 Group: *P* = 0.876
	UBROW	−19.0 (−37.0 to 1.1)^a^	−4.1 (−20.6 to 12.4)	−14.0 (−31.4 to 3.4)		
Max ΔDBP (mmHg))	CON	−7.2 (−18.7 to 4.4)	−3.2 (−15.2 to 8.8)	−12.2 (−24.7 to 0.4)	0.023	Time × group: *P* = 0.759 Time: *P* = 0.044 Group: *P* = 0.731
	UBROW	−8.4 (−19.1 to 2.3)^a^	0.9 (−9.5 to 11.3)	−8.9 (−19.7 to 2.0)		
Incidence of OH (*n*/total sample (%))	CON	3/6 (50%) *P* = 1.000	2/6 (33.3%) *P* = 1.000	2/6 (33.3%) *P* = 1.000		
	UBROW	4/7 (57.1%)	3/8 (37.5%)	2/8 (25%)		

Data are presented as means (95% confidence intervals). ^a^ *n* = 7 in UBROW due to missing value for one subject. Note that for all blood pressure values, *n* = 6 in CON. Incidence of OH (CON vs. UBROW) analysed by Fisher's exact test. CON, control group; DBP, diastolic blood pressure; OH, orthostatic hypotension; SBP, systolic blood pressure; UBROW, upper‐body rowing exercise group.

## 4 DISCUSSION

In this study, 12 weeks of UBROW did not result in any changes in HRV or incidence of OH, or HR_peak_ obtained during a cardiopulmonary exercise test, in individuals with chronic SCI. Whilst 12 weeks of UBROW enlarged brachial artery lumen size, there were no changes in carotid artery dimensions (lumen and wall thickness), suggesting local (and not systemic) remodelling of arteries in response to 12 weeks of volitional upper‐body exercise adhering to current exercise guidelines.

### 4.1 Autonomic cardiovascular responses to the exercise intervention

In line with our findings, Dorey et al. (2024) recently reported no effects of either 6 months of arm‐cranking exercise or body‐weight supported treadmill training on blood pressure responses to a sit‐up test or the incidence of OH, in individuals with motor‐complete high‐level SCI (NLI: C4–T6). In non‐injured individuals, sympathetic‐induced increases in vascular resistance in response to baroreceptor unloading offset the immediate reduction in arterial blood pressure by maintaining cardiac output and total peripheral resistance (Ogoh et al., 2003; Smit et al., 1999). In individuals with high‐level SCI, disrupted sympathetic innervation of the vasculature may not counter such orthostatic‐induced blood pooling in the lower‐body's capacitance veins through vasoconstriction, leading to reductions in cardiac preload, stroke volume and cardiac output, and ultimately OH (Claydon & Krassioukov, 2006). Therefore, among individuals with high‐level SCI, orthostatic tolerance is likely reliant on efficient parasympathetic withdrawal that increases heart rate to support blood pressure. Solinsky et al. (2021) reported that high‐intensity hybrid FES rowing enhanced cardiovagal baroreflex sensitivity as measured by the neck suction technique in individuals with SCI. Similarly, Dorey et al. (2024) found in their cohort of individuals with high‐level SCI that arm‐cranking exercise training resulted in improved cardiovagal baroreflex sensitivity alongside reductions in HF power HRV responses to the sit‐up test, indicating enhanced parasympathetic regulation following the exercise intervention. In contrast, we did not find any effects of training on the autonomic regulation of heart rate via time and frequency domain analysis of HRV. However, it should be considered that we measured HRV at rest and not during the sit‐up test (Dorey et al., 2024). Moreover, the discrepancy between the present findings and Dorey et al. (2024) could also be due to the difference in the length of interventions (12 weeks vs. 6 months), or due to the different injury characteristics of the participants in the two studies (high‐level motor‐complete SCI in Dorey et al. vs a rather heterogeneous injury characteristics of our cohort). Heterogeneity among the participants in our study may possibly have resulted in varied exercise stimuli (e.g. through variation in absolute metabolic demand due to differences in motor‐function) (Hutchinson & Goosey‐Tolfrey, 2022) as well as inter‐individual differences in the ability to adapt to exercise (West, 2023).

Nonetheless, some noteworthy observations were evident in the individual data of one of the two cervical‐injured participants in the UBROW group. This participant (NLI: C6, AIS: B) was the only participant unable to increase exercise heart rate above 130 bpm (indicating autonomic complete SCI; West, Romer et al., 2013) and demonstrated pre‐ to post‐exercise reductions in HF power and RMSSD, and an increase in mean heart rate, which are consistent with reductions in cardiac parasympathetic tone (Shaffer & Ginsberg, 2017). Interestingly, these changes translated into better orthostatic tolerance, as the OH that occurred at baseline was absent at post‐intervention for this participant. Such a potential beneficial effect of upper‐body rowing on orthostatic tolerance through vagal inhibition in cervical SCI needs to be further explored.

### 4.2 Vascular adaptations to the exercise intervention

A novel aspect of the current study was the assessment of both central and peripheral artery dimensions in response to upper‐body exercise training. Consistent with previous cross‐sectional observations in SCI (Huonker et al., 2003), and longitudinal reports in non‐injured individuals (Dinenno et al., 2001; Miyachi et al., 2001), we demonstrated that exercise training was associated with localized arterial adaptation, as shown by a significant increase in brachial artery diameter immediately post‐intervention. A potential mechanism for the localized adaptation in arterial diameter may relate to the shear stress stimulus acting on the vascular endothelium, as adaptations in brachial artery function and size after handgrip exercise training in non‐injured subjects were abolished by reducing exercise‐induced shear stress (Tinken et al., 2010). While previous studies have interpreted resting conduit artery diameter as an indicator of artery structure (Dinenno et al., 2001; Miyachi et al., 2001; Schmidt‐trucksa et al., 2000), it is important to note that several factors may influence resting diameter through changes in vascular tone, including circulating hormone modulation, local endothelium‐derived factors and sympathetic outflow (Naylor et al., 2005). Whilst we did not account for all these potential influences, our data did not indicate any changes in sympathetic activity pre‐post exercise (as inferred by HRV, blood pressure response to the sit‐up test, and HR_peak_), indicating that changes in vascular tone (i.e. reduced vasoconstriction) did not explain the significant enlargement of the brachial artery. This is further supported by the lack of correlation between changes (baseline to 12 weeks) in brachial artery diameter and changes in HRV outcomes. Hence, the increased arterial diameter may likely reflect structural remodelling similar to observations in non‐injured individuals who demonstrate localized structural arterial enlargement in the trained limb following exercise training (Tinken et al., 2008).

Our data indicate no effect of UBROW on carotid lumen size or wall thickness, which is consistent with similar exercise training studies showing no effect on carotid artery dimensions in individuals with SCI (CIMT, wall‐to‐lumen ratio, Totosy de Zepetnek et al., 2015; resting cross‐sectional area, Ditor, MacDonald et al., 2005; or carotid‐femoral pulse wave velocity, Alrashidi et al., 2021). In contrast, previous cross‐sectional studies have reported a smaller CIMT and carotid artery wall‐to‐lumen ratio in physically active versus sedentary individuals with SCI (Matos‐Souza et al., 2013; Rowley et al., 2012), and similar CIMT and wall‐to‐lumen ratio between trained athletes with SCI and recreationally active, non‐injured individuals (Matos‐Souza et al., 2013). Several factors may explain the lack of change in carotid artery dimensions. As suggested previously by Thijssen et al. (2012), modification of carotid wall thickness may require prolonged or intense exercise exposure exceeding the typical 12‐ to 24‐week‐long interventions adopted in many SCI exercise studies (Alrashidi et al., 2021; Hansen, Samani, Laessoe et al., 2023; Totosy de Zepetnek et al., 2015). The lack of change in CIMT and carotid diameter in the current study could also be due to the varying haemodynamic stimuli originating from the rowing exercise, which included a combination of resistance and aerobic training that may have had opposing effects on the arterial wall. In non‐injured individuals, aerobic training has been shown to reduce arterial stiffness (Seals et al., 2008), whereas resistance training has been shown to have no impact (Poelkens et al., 2007) or increase arterial stiffness (Bertovic et al., 1999). However, as rowing exercise has been shown to negate the stiffening effects of resistance training on arterial compliance in healthy, non‐injured rowers (Cook et al., 2006), such an explanation seems unlikely. Alternatively, exercising with the limited muscle mass of the upper‐body may have provided inadequate stimulus for systemic vascular adaptations (e.g. central artery dimensions).

### 4.3 Limitations

Some limitations of this study should be acknowledged. The relatively small, heterogeneous sample size of the study is suboptimal; however, small sample sizes are not uncommon in similar longitudinal exercise studies in this population (e.g. Ditor, Kamath et al., 2005; Ditor, MacDonald et al., 2005; Dorey et al., 2024) due to the lack of availability of people with SCI. The relatively small sample size and inclusion of participants with a wide range of neurological level of injuries did not allow for separate (adequately powered) analyses of paraplegic and tetraplegic participants or dichotomizing by injuries above and below T6 to account for differences in retained sympathetic cardiovascular function. In our study, we assessed HRV during spontaneous breathing. Although this approach has been commonly used, a recent study in SCI indicates that respiration variability directly influences HRV, and concluded that respiration, therefore, should be controlled by assessing HRV during paced breathing (Solinsky et al., 2022). Also, measuring HRV during the sit‐up test could have added additional insights into autonomic cardiac responses to orthostatic stress. Finally, we employed a test battery of several indirect measurements of autonomic cardiovascular control. Despite the novelty of this approach in SCI exercise studies, we could have performed a more direct and comprehensive evaluation of ANS function using a modified autonomic reflex screen test battery, including measurements of sympathetic skin responses, heart rate responses to deep breathing, and blood pressure responses to the Valsalva manoeuvre (Berger et al., 2017).

### 4.4 Conclusion

We found no evidence of changes in HRV, or blood pressure responses to a sit‐up test following 12 weeks of upper‐body rowing exercise, suggesting that the exercise intervention did not modulate autonomic cardiovascular control in individuals with chronic SCI. Notably, our results provide novel evidence demonstrating that 12 weeks of volitional upper‐body exercise, following current guidelines, resulted in localized enlargement of the brachial artery diameter with no influence on carotid artery dimensions (diameter and CIMT).

## AUTHOR CONTRIBUTIONS

Conception or design of the work: Rasmus Kopp Hansen, Stefanos Volianitis, Uffe Laessoe, Afshin Samani, and Ryan Godsk Larsen. Acquisition or analysis or interpretation of data for the work: Rasmus Kopp Hansen, Rasmus Bering, Claus Graff, Stefanos Volianitis, and Ryan Godsk Larsen. Drafting the work or revising it critically for important intellectual content: Rasmus Kopp Hansen, Rasmus Bering, Claus Graff, Stefanos Volianitis, Uffe Laessoe, Afshin Samani, and Ryan Godsk Larsen. All authors approved the final version of the manuscript and agree to be accountable for all aspects of the work in ensuing that questions related to the accuracy or the integrity of any part of the work are appropriately investigated and resolved. All persons designated as authors qualify for authorship, and all those who qualify for authorship are listed.

## CONFLICT OF INTEREST

None declared.

## Data Availability

The datasets of the current study are available from the corresponding author upon reasonable request.
